# Asthma routinization, family asthma management, caregiver depressive symptoms, and medication adherence in Head Start preschool children

**DOI:** 10.3389/falgy.2023.1219868

**Published:** 2023-09-28

**Authors:** Monica A. Lu, Elizabeth Ruvalcaba, Elizabeth L. McQuaid, Cynthia S. Rand, Kristin A. Riekert, Michelle N. Eakin

**Affiliations:** ^1^ Eudowood Division of Pediatric Respiratory Sciences, Department of Pediatrics, Johns Hopkins School of Medicine, Baltimore, MD, United States; ^2^ Division of Pulmonary and Critical Care Medicine, Department of Medicine, Johns Hopkins School of Medicine, Baltimore, MD, United States; ^3^ Department of Psychiatry and Human Behavior, Alpert Medical School, Brown University, Providence, RI, United States; ^4^ Department of Pediatrics, Alpert Medical School, Brown University, Providence, RI, United States

**Keywords:** routines, family management, medication adherence, caregiver depression, childhood asthma

## Abstract

**Introduction:**

Medication adherence is suboptimal in childhood asthma. Children rely on caregivers to manage medication administration. It is important to detect families who are at risk for poor adherence or to identify potential areas that can assist families with better adherence to asthma medications in order to improve asthma outcomes. We investigated the association between asthma routines, family asthma management knowledge and skills, and caregiver depressive symptoms with daily controller medication adherence among Head Start preschool children in Baltimore City.

**Methods:**

Our study included 256 low-income urban preschool children who were prescribed a daily controller medication. Asthma routinization (by the Asthma Routines Questionnaire), family asthma management [by the Family Asthma Management System Scale (FAMSS)], and caregiver depressive symptoms (by the Center for Epidemiological Studies – Depression) were assessed at baseline. The medication possession ratio (MPR) to measure adherence to daily controller medications was calculated at baseline and 12 months from pharmacy fill records. Multiple regression models evaluated the relationship between asthma routinization, the FAMSS, the CES-D, and MPR.

**Results:**

Results indicated that only 7% of families had an MPR above 80% at baseline, and 24% of caregivers had clinically significant depressive symptoms. Higher asthma medication routines were associated with higher MPR at baseline (*b* = 0.05, *p* = 0.03). Higher family asthma management was associated with higher MPR at both baseline (*b* = 0.04, *p* < 0.01) and 12 months (*b* = 0.05, *p* < 0.01).

**Discussion:**

Our findings highlight the importance of family asthma management and maintaining medication routines over time to improve asthma controller medication adherence.

## Introduction

Asthma disproportionately affects minority low-income children, with high rates of morbidity and mortality from asthma in this group (1). Low rates of both measured and self-reported adherence to daily controller medications, such as inhaled corticosteroids, are reported in urban minority children (2–5). The preschool age group in particular has the most asthma-related healthcare visits compared to older children (6).

In the preschool age group, families and caregivers bear the primary responsibility for asthma care and medication management. Asthma management affects all family members living in the household, and caregivers must establish effective strategies to maintain and treat their child's asthma. Strategies utilized can include incorporating daily management of medications into family routines, as well as ensuring family integration of asthma management into their daily lives (7). Asthma controller administration requires a daily routine and pattern with regularity, set timing, and planning for use of the child's medication, but that continued management may also have families perceiving asthma medication management as a hassle or chore.

Caring for a child with asthma also affects the mental health of caregivers. Depression was shown to be more common in caregivers of children with asthma than caregivers of healthy children in a meta-analysis (8). Caregiver depression symptoms has also been associated with more asthma symptoms in the child, including fewer symptom-free days and decreased medication adherence (9–12). In addition, caregiver depressive symptoms can impact the ability to participate in asthma management skills or develop daily routines. Previous studies have shown better family asthma management was inversely correlated with asthma morbidity, and families with better medication routines for their children demonstrate better medication adherence (13–15).

In this study, we evaluated the association between asthma routines, family asthma management, and caregiver depressive symptoms with medication adherence to daily controller medications, which has not previously been examined among low-income urban children in Baltimore City Head Start preschool programs. The outcomes included medication adherence using medication possession ratio (MPR) calculated at baseline and at 12 months. We hypothesized that higher asthma routinization, a higher score for family asthma management, and fewer caregiver depressive symptoms would be associated with higher medication adherence at both baseline and 12 months in this urban minority preschool group.

## Methods

### Participants

Participants were recruited from Baltimore City Head Start programs across 78 sites from April 2011 to November 2016 as part of a randomized trial of a multi-level home and school-based asthma education program (16). Eligible caregivers were the parent or legal guardian of a child aged 2–years, who reported that their child had a physician diagnosis of asthma or reactive airway disease, and who spoke English. Inclusion criteria for this study's subgroup analysis included being prescribed a daily controller medication by their health care provider and availability of pharmacy fill data.

### Procedures

The Johns Hopkins School of Medicine Institutional Review Board approved the study, and informed consent was obtained prior to any data collection. Research assistants conducted home visits at baseline, which included a structured survey with the caregiver. Families in the study were randomized to either receive a family education intervention (Asthma Basic Care; ABC) with Head Start education (ABC + HS) or Head Start education alone (16). The ABC family intervention included in person and at home asthma management educational sessions and phone calls tailored to the needs of preschool children living in urban home environments.

### Measures

#### Caregiver depressive symptoms (center for epidemiological studies depression scale)

Caregiver depressive symptoms were assessed using the 20-item Center for Epidemiological Studies Depression scale (CES-D). The CES-D measures the level of depression symptomatology at the time of questionnaire administration and asks about the frequency of symptoms during the past week. Responses range from 0 (Rarely or none of the time—less than 1 day), (1) (Some or a little of the time – 1–2 days), (2) (Occasionally or a moderate amount of the time – 3–4 days), or 3 (Most or all of the time – 5–7 days) (17). Four items related to well-being are reverse coded. A total score is obtained with higher scores indicating greater depressive symptoms. A score of 16 or greater is also considered suggestive of individuals at risk for clinical depression (17).

#### Asthma routinization (asthma routines questionnaire)

The Asthma Routines Questionnaire measures asthma management routinization, and includes two subscale scores *Medication Routines* and *Routine Burden* (14). *Medication Routines* is related to regularity of medications, predictability, appointment planning, and pharmacy refills, and *Routine Burden* is related to caregiver and child quality of life and whether asthma management is perceived as a chore or burden. Each of the 8-items had scores ranging from 1 to 4. A higher *Medication Routines* score indicates better medication routines, and a higher *Routine Burden* score indicates more perceived burden with management routines.

#### Family asthma management system scale (FAMSS)

The Family Asthma Management System Scale (FAMSS) is a semi-structured interview to assess family management of pediatric asthma and can be administered to caregivers of young children (13, 18, 19). The interview is recorded and rated using a standard manual with 7 subscales which assess key areas of asthma management: (1) *Asthma Knowledge*, (2) *Symptom Assessment*, (3) *Family Response to Exacerbations*, (4) *Environmental Control*, (5) *Medication Adherence*, (6) *Collaborative Relationship with Health Care Provider*, and (7) *Balanced Integration of Asthma and Family Life***.** Subscales are rated 1 to 9, with the FAMSS *Total Score* calculated as the mean of the ratings on the constituent subscales. Higher scores indicate better asthma management. A description of the FAMSS interview and reliability for this study population, as well as further information about the FAMSS subscales, was published previously (19).

#### Medication possession ratio (MPR) for daily controller medications

A Current Medication Treatment Plan (CMTP) was obtained from each child's primary care provider. The CMTP contained information about daily controller asthma medications including drug name, dose, frequency, and medication start and end dates. Medication refill data was obtained by faxing all family-identified pharmacies to request refill records. A Medication Possession Ratio (MPR) is an objective measure for adherence to daily controller medications in asthma. MPR for a daily controller medication is defined as the days supply of medication dispensed by a pharmacy (obtained by medication refill data from pharmacies) divided by the number of days the child was expected to be receiving the medication (obtained from the CMTP) and ranged from 0 to 1.00. The MPR intervals were calculated as 12 months prior to the date of the baseline for the baseline MPR and 12 months forward from the date of the baseline for the 12 month MPR. If a medication was started or discontinued during the interval, these dates were used. The MPRs of each medication were averaged across all controller medications to obtain the composite MPR. If a child was prescribed only one drug, then the composite MPR would be equal to his or her MPR for that individual drug. Daily asthma controller medications included: inhaled corticosteroids, combination inhaled corticosteroids and long-acting beta 2 agonists, and montelukast.

#### Test for respiratory and asthma control in kids (TRACK)

The Test for Respiratory and Asthma Control in Kids (TRACK) was completed by the caregiver to evaluate degree of asthma control. The TRACK has been validated in preschool children with asthma (20). 5 standardized questions address risk and impairment factors, including frequency of wheeze, nighttime awakenings, activity limitation, use of rescue medications, and oral corticosteroid use in the past year. Each question was scored using a 0–20 Likert scale for a total score range of 0–100. A binary cutpoint of TRACK less than 80 indicated that asthma was uncontrolled for regression models (20).

### Statistical analyses

Descriptive statistics for the Asthma Routines Questionnaire, FAMSS, and CES-D were calculated with means, standard deviations, and ranges at baseline, and also for MPR for daily controller medications at baseline and 12 months. The relationships between asthma routines, family asthma management, and medication possession ratio were studied using Pearson's correlation coefficients in a correlation matrix, with the FAMSS subscales added post-hoc. Individual multiple regression models were conducted to evaluate the association of the two Asthma Routines Questionnaire subscales (*Routine Burden* and *Medication Routine*), the FAMSS Total Score, caregiver depressive symptoms (measured by the CES-D total score), and the outcomes of MPR at baseline or 12 months. After the model was run with the FAMSS Total Score, a post-hoc exploratory model was run to examine which specific FAMSS subscale scores were associated with MPR. Models were controlled for *a priori* selected covariates of family history of asthma, number of people living in the household, the number of daily controller medications, and asthma control (based on the TRACK score <80 for uncontrolled asthma). Models at 12 months also controlled for the intervention group (Head Start education alone or ABC + HS) (16). All analyses were conducted in Stata/MP 16.0 (StataCorp, College Station, TX).

## Results

**Figure 1:**
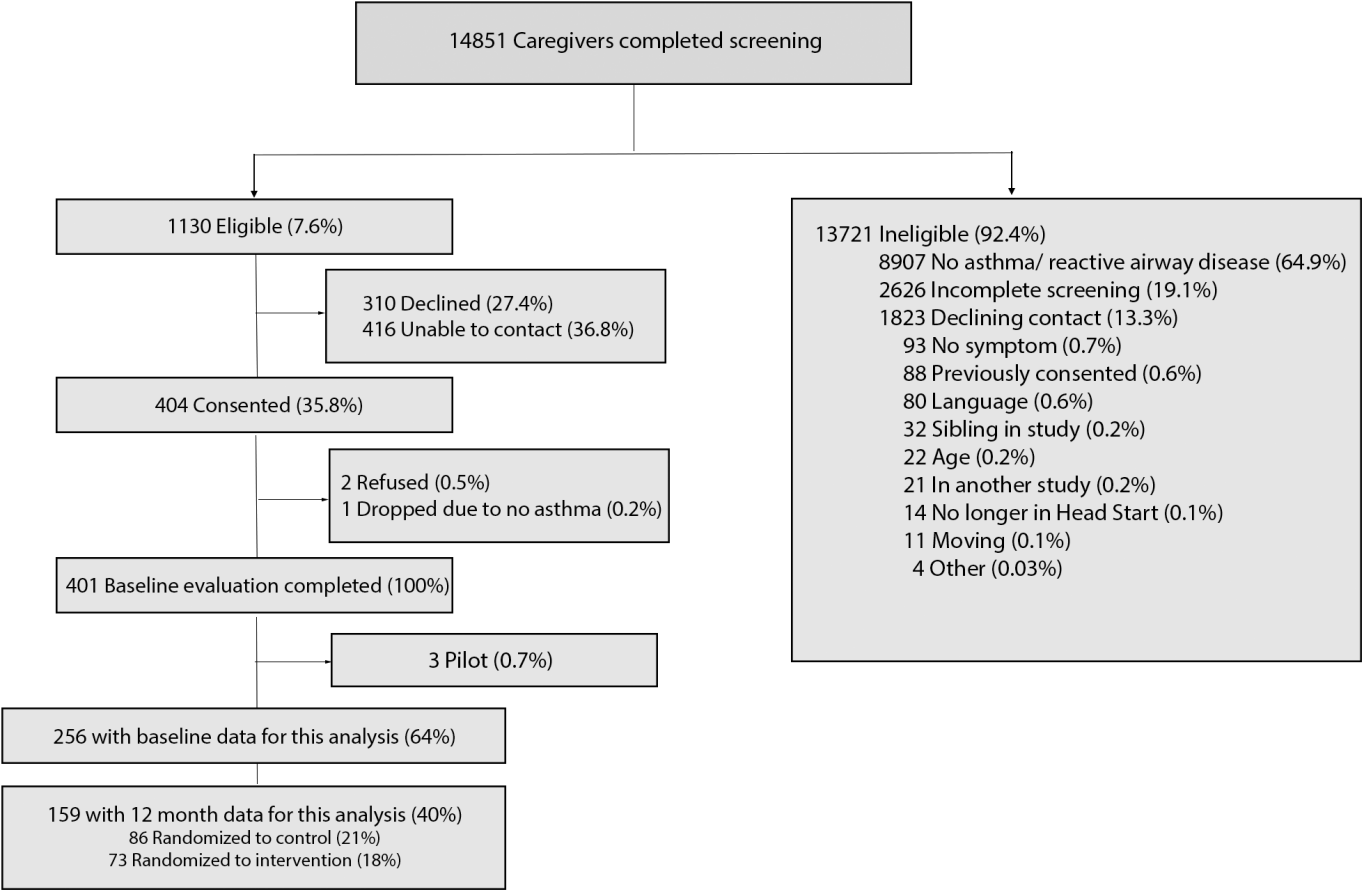
Consort diagram.

Fourteen thousand eight hundred fifty-one caregivers (90% of the children enrolled at Head Start) completed screening between 2012 and 2016 (Figure 1). 24% of these children had reported asthma diagnoses. One thousand one hundred thirty (8%) were eligible and provided permission to be contacted for the previously described Asthma Basic Care study (16). Four hundred four caregivers were consented. For this subgroup analysis, 256 children were eligible at baseline; eligibility included being prescribed a daily controller medication by their primary care provider and availability of pharmacy fill data.

### Descriptive statistics and correlation matrix

**Figure 2:**
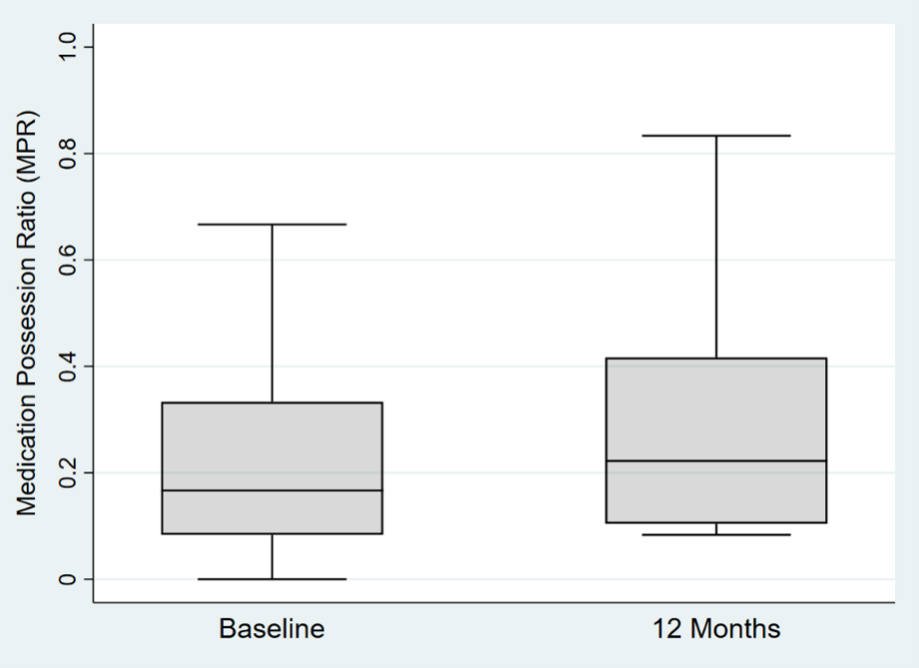
Box plot of medication possession ratio (MPR) at baseline and 12 months.

The mean age of enrolled children at the baseline assessment in this analysis was 4.2 years old (SD = 0.7), and 251 (98%) of children were Black/African-American. 198 (77%) had a household income below $30,000 per year, and 243 (96%) received public insurance at baseline (Table 1). 176 (69%) of children had uncontrolled asthma (TRACK score <80) and 76 (30%) were prescribed two daily controller medications. 59 (24%) of caregivers screened positive for elevated depressive symptoms on the CES-D (score 16 or greater). 203 (79%) had a family history of asthma and 115 (47%) had more than 5 people living in the household. Median MPR at baseline and 12 months was 0.17 and 0.22 respectively, see Figure 2.

**Table 1:** Baseline characteristics of children and caregivers.

Characteristic	Baseline *n* (%) (*n* = 256)
Child characteristics	
Age in years, *mean (sd)*	4.2 (0.7)
Sex	
Male	169 (66)
Female	87 (34)
Race	
Black/African-American	251 (98)
White (non-hispanic)	6 (2)
Hispanic	5 (2)
Health Insurance	
Public insurance	243 (96)
Private insurance	6 (2)
Unknown or uninsured	3 (1)
Uncontrolled asthma (TRACK score <80)	176 (69)
Family history of asthma	203 (79)
Number of people who live in the household	
2–4 household members	138 (53)
5 or greater household members	115 (47)
Number of daily controller medications	
1 daily controller medication	171 (67)
2 daily controller medications	76 (30)
Caregiver characteristics	
Age in years, *mean (sd)*	31.9 (8.6)
Relationship to child	
Mother/stepmother	223 (87)
Father/stepfather	16 (6)
Grandmother	9 (4)
Other	8 (3)
Education	
Some high school completed	44 (17)
High school graduate or GED	94 (37)
Some college or trade school	95 (37)
College graduate	22 (9)
Low household income (<$30,000)	198 (77)
Elevated caregiver depressive symptoms (CES-D score 16 or greater)	59 (24)

The Asthma Routines Questionnaire subscales, FAMSS Total Score and subscale scores, and CES-D are described with means and standard deviations at baseline. The Medication Possession Ratio is described at baseline and 12 months (Table 2). A correlation matrix showed weak to strong correlations between variables. The highest correlations were noted between the FAMSS *Total Score* and FAMSS subscale scores. Weak to moderate correlations were noted between medication adherence and medication routines and FAMSS *Total Score*.

**Table 2:** Means, standard deviations, and correlation matrix.

Variables	Mean	SD	1	2	3	4	5	6	7	8	9	10	11	12
1. Asthma Routines Questionnaire Subscale - Routine Burden at Baseline	2.93	0.77	1											
2. Asthma Routines Questionnaire Subscale - Medication Routine at Baseline	3.18	0.69	−0.05	1										
3. Family Asthma Management System Scale Total Score at Baseline	4.88	1.27	−0.04	0.24**	1									
4. FAMSS Subscale: Asthma knowledge at Baseline	4.89	1.72	−0.07	0.26**	0.68**	1								
5. FAMSS subscale: Symptom assessment at Baseline	4.87	1.54	−0.16**	0.24**	0.63**	0.58**	1							
6. FAMSS subscale: Family response at Baseline	5.28	1.91	−0.03	0.16*	0.80**	0.50**	0.52**	1						
7. FAMSS subscale: Environmental control at Baseline	3.53	2.30	0.05	0.01	0.52**	0.09	0.08	0.21**	1					
8. FAMSS subscale: Medication adherence at Baseline	5.59	1.93	−0.02	0.19**	0.71**	0.37**	0.30**	0.55**	0.24**	1				
9. FAMSS subscale: Collaboration at Baseline	5.20	1.80	−0.05	0.19**	0.73**	0.46**	0.38**	0.62**	0.13*	0.50**	1			
10. FAMSS subscale: Balanced integration at Baseline	4.80	1.77	0.07	0.16*	0.75**	0.37**	0.33**	0.46**	0.50**	0.44**	0.46**	1		
11. Caregiver Depressive Symptoms (CES-D) at Baseline	11.74	9.37	−0.07	−0.09	−0.21**	−0.13*	−0.01	−0.09	−0.24**	−0.12	−0.16*	−0.21**	1	
12. Medication Possession Ratio at Baseline	0.24	0.24	−0.12	0.21**	0.27**	0.25**	0.14*	0.19**	0.12	0.17**	0.28**	0.18**	−0.04	1
13. Medication Possession Ratio at 12 months	0.27	0.21	−0.01	0.06	0.23**	0.16*	0.08	0.15	0.16*	0.18*	0.21**	0.11	0.03	0.37**

A higher asthma routines questionnaire routine burden score indicates more perceived burden with management routines, and a higher medication routine score indicates better medication routines. A higher family asthma management system scale indicates better family asthma management; higher individual subscale scores indicate better family management for that area of asthma management. The Center for Epidemiological Studies Depression Scale (CES-D) score ranges from 0 to 60 with higher scores indicating more depressive symptoms. The medication possession ratio ranges from 0 to 1.00.

* *P* < 0.05.

** *P* < 0.01.

### Association of the asthma routines questionnaire, FAMSS, and caregiver depressive symptoms

Consistent with our hypothesis, higher *Medication Routines* scores were significantly associated with higher MPR at baseline (*b* = 0.05, 95% CI = 0.004–0.09, *p* = 0.03); however, this association was not seen at 12 months (Table 3). In addition, higher FAMSS *Total Scores* were significantly associated with higher MPR at both baseline (*b* = 0.04, 95% CI = 0.02–0.07, *p* < 0.01) and 12 months (*b* = 0.05, 95% CI = 0.02–0.08, *p* < 0.01). Contrary to our hypotheses, caregiver depressive symptoms and the *Routine Burden* subscale of the Asthma Routines Questionnaire were not associated with MPR at either baseline or 12 months.

**Table 3:** Multiple regression models for baseline asthma routine questionnaire subscales, FAMSS, and caregiver depressive symptoms and the outcome of MPR at baseline and 12 months.

Variable	Baseline - MPR			12 months - MPR		
	Coefficient	*P* value	95% CI	Coefficient	*P* value	95% CI
Asthma routines questionnaire						
Routine burden	−0.03	0.18	−0.06, 0.01	−0.007	0.77	−0.05, 0.04
Medication Routines	0.05	0.03*	0.004, 0.09	0.004	0.86	−0.05, 0.06
FAMSS total score	0.04	<0.01**	0.02, 0.07	0.05	<0.01**	0.02, 0.08
Caregiver Depressive Symptoms (CES-D)	0.0003	0.80	−0.003, 0.004	0.002	0.38	−0.002, 0.01

Regression models are adjusted for asthma control, family history of asthma, the number of people living in the household, and the number of daily controller medications. Outcomes at 12 months are also controlled for the ABC intervention group.

* *P* < 0.05.

** *P* < 0.01.

As the FAMSS Total Score was associated with MPR at baseline and 12 months, post-hoc analyses were run to determine which specific FAMSS subscale scores were associated with MPR (Table 4). Higher *Asthma Knowledge* (*b* = 0.02, 95% CI = 0.002–0.05, *p* = 0.03) and *Collaborative Relationship with Health Care Provider* subscale scores (*b* = 0.02, 95% CI = 0.002–0.05, *p* = 0.03) were significantly associated with higher MPR at baseline. Higher *Environmental Control* subscale scores were significantly associated with higher MPR at 12 months (*b* = 0.02, 95% CI = 0.002–0.04, *p* = 0.03). Higher *Medication Routines* scores remained significantly associated with higher MPR at baseline (*b* = 0.04, 95% CI = 0.0003–0.09, *p* = 0.048), but not at 12 months.

**Table 4:** Post-hoc multiple regression models for baseline asthma routine questionnaire subscales, FAMSS subscales, and caregiver depressive symptoms and the outcome of MPR at baseline and 12 months.

Variable	Baseline - MPR			12 months - MPR		
	Coefficient	*P* value	95% CI	Coefficient	*P* value	95% CI
Asthma routines questionnaire						
Routine burden	−0.03	0.18	−0.06, 0.01	−0.01	0.65	−0.06, 0.04
Medication routines	0.04	0.048*	0.0003, 0.09	0.004	0.90	−0.05, 0.06
FAMSS Subscales						
Asthma knowledge	0.02	0.03*	0.002, 0.05	0.02	0.27	−0.01, 0.04
Symptom assessment	−0.009	0.44	−0.03, 0.01	−0.01	0.39	−0.04, 0.02
Family response	−0.007	0.55	−0.03, 0.02	−0.002	0.99	−0.03, 0.03
Environmental control	0.008	0.26	−0.01, 0.02	0.02	0.03*	0.002, 0.04
Medication adherence	0.0004	0.96	−0.02, 0.02	0.02	0.21	−0.01, 0.04
Collaboration	0.02	0.03*	0.002, 0.05	0.02	0.21	−0.01, 0.05
Balanced integration	0.004	0.69	−0.02, 0.03	−0.007	0.60	−0.03, 0.02
Caregiver Depressive Symptoms (CES-D)	0.001	0.53	−0.002, 0.004	0.002	0.27	−0.001, 0.01

Regression models are adjusted for asthma control, family history of asthma, the number of people living in the household, and the number of daily controller medications. Outcomes at 12 months are also controlled for the ABC intervention group.

* *P* < 0.05.

## Discussion

Our study demonstrated medication routines and family asthma management are associated with higher medication adherence to daily asthma controller medications in a low-income urban preschool group. There remained a continued association between higher family management of asthma at baseline and higher medication adherence to daily controller medications at 12 months. However, these associations were seen in the context of overall poor adherence to daily controllers both at baseline and 12 months in this study population, with only 7% of families with an MPR above 80% at baseline. This study did uniquely examine objective measures of asthma controller adherence and how family medication routines and asthma management knowledge and skills influence adherence in a high-risk urban preschool population. As caregivers and family members manage asthma for preschool children, these results emphasize the importance of supporting families in developing medication routines and understanding asthma management skills and knowledge in order to optimize adherence and subsequent asthma outcomes. Furthermore, while caregiver depression was not associated with medication adherence in this sample, there were very high rates of depressive symptoms which suggest a need for monitoring as well.

This study stresses the importance of strong medication routines and their effects on medication adherence. Families need to successfully incorporate their child's asthma medication management into daily routines. This may occur through the development of habits, which can form from repeated behavior in a specific context or setting, and repeating that behavior or strengthening that habit may increase how automatic the behavior becomes (21, 22). As habits become more automatic and integrated into behavior, they may override other desires or other conscious factors that come up and may also predict adherence to certain actions, such as administration of daily medications. These formed habits may contribute to medication routines and may even overcome any perceived burden or feeling that an action is a chore, as the habits become automatic. Habit strength has been previously associated with higher medication adherence rates among chronic diseases, including with inhaled corticosteroids for asthma in older populations, so assessing habit strength in addition to medication routines in future research may aid in understanding of adherence (23–25).

The effect of medication routines on medication adherence was seen at baseline, but not at 12 months. In order for medication routines to be effective, they need to be maintained by the family and caregivers. The difference in timeframe of 12 months and likely changes in a child's developmental stages during this time may have affected this association being seen at baseline and not at 12 months. The association of medication adherence with family asthma management skills did remain consistent both at baseline and 12 months, highlighting the interplay and importance of the family in maintaining these routines and adherence. Our previous study had also demonstrated that better overall family asthma management and being able to balance the integration of asthma management and family life were associated with fewer oral steroid courses required in this same population (19). In post-hoc exploratory analyses for this study, individual FAMSS subscale scores were associated with medication adherence, including *Asthma Knowledge* and *Collaborative Relationship with Health Care Provider* at baseline and *Environmental Control* at 12 months. Examining these specific areas for family education in the future may help with targeting adherence for families. The difference in associations seen with medication routines and family asthma management may be due to a measurement methodology for the scales; *Medication Routines* were self-reported, while the FAMSS was scored by researchers based on open-ended responses or descriptions provided by the family.

Surprisingly, caregiver depressive symptoms were not associated with medication adherence, as previous studies had showed caregiver depression to be associated with worse medication adherence (10, 11). Depressive symptoms are likely linked to a number of other socio-ecological risk factors or stressors that were not examined in these analyses and affect medication adherence, which may mask an association being seen with adherence (26). Nearly half of our population also had five or greater household members living in the home, and depression was only screened in the primary caregiver and not in other household or family members who may be involved in asthma care. The number of household members may contribute to day-to-day variation in who has asthma knowledge, administers daily medications, speaks to the healthcare provider, and manages the child's asthma, though we did control for the number of people living in the household as a covariate. On the other hand, the associations seen with the FAMSS interview may have assessed other household members beyond the primary caregiver, as it rated the overall quality of the asthma management, regardless of how many family members or who provides the care. Furthermore, objective measures of adherence were low in this population, with adherence with pharmacy fill data at around one-quarter of the time (mean MPR at baseline was 0.24 and mean MPR at 12 months was 0.27), so this may have affected the association seen. Caregiver depression is still important for clinicians to evaluate, especially in this population where 24% of caregivers screened positive for depression. However, our results suggest that interventions to support development of medication routines and habits may even be effective in families with high levels of caregiver depressive symptoms.

There were limitations in this study. First, the medication possession ratio (MPR) measures whether a medication was filled at a pharmacy and not whether the child had taken the medication. However, objective measures of pharmacy fill data are still likely more reliable than self-report by patients or caregivers, which may overestimate adherence compared to pharmacy fill data in asthma and other chronic diseases (27, 28). Second, participants in this study were randomized to receive either a family asthma education intervention or to a control group. The intervention group was controlled for as a covariate in our analyses at 12 months. Being randomized to the intervention group may have affected how supported a family felt during the study with frequent check-ins and scheduled education and that may have affected adherence over time. The CONSORT diagram delineates the number of participants randomized to the intervention at 12 months. Third, the CES-D screens for depression captures symptoms over the prior one-week period and may not have captured caregiver symptoms over the entire 12 month period measured by MPR. Lastly, this study data was collected between 2011 and 2016, which was prior to the COVID pandemic and may not reflect impacts of COVID.

Our results demonstrate that medication routines and family asthma management may play a more important role than routine burden in supporting medication adherence. As children grow older and progress through developmental stages, these routines may continually adjust and change. Clinicians should consistently reassess medication routines and encourage the development of good habits which have been shown here to directly affect adherence to medications. Behavioral interventions that help families strengthen the development of consistent routines may be important to improve medication adherence. These results emphasize that these interventions to support the development of routines may be important even in a population with high levels of caregiver depressive symptoms, but also highlight that continued support over time is needed in order to ensure optimal adherence long term.

## Data Availability

The data analyzed in this study is subject to the following licenses/restrictions: Containing information that could compromise the privacy of research participants. Requests to access these datasets should be directed to meakin1@jhmi.edu.
